# Supplementary material for: Spatiotemporal Localisation of Heparan Sulphate Proteoglycans throughout Mouse Lens Morphogenesis
Source: Cells. 2023 May 11;12(10):1364. doi: 10.3390/cells12101364 (PMC10216587; doi:10.3390/cells12101364)
Supplement: Supplementary file 1 [file cells-12-01364-s001.zip › cells-2381133-supplementary.pdf]

Table S1. Primary antibodies used for GAG and core protein immunolocalisation.

	Target	Antibody
**Sulfation Enzyme**	PAPSS2	pAb, Rb IgG<br>Abcam, Cat#: ab155588
**Sulfated GAGs**	Heparan sulfate (HS-GAG)	mAb (F58-10E4) Ms IgMκ<br>Amsbio, Cat#: 370255-1
	Chondroitin Sulfate (CS-GAG)	mAb (CS-56), Ms IgM<br>Sigma, Cat#: C8035
**HSPG Core Protein**	Syndecan-1 (CD138)	pAb, Ms IgG1κ<br>Santa Cruz Biotechnology, Cat#: sc-12765
	Syndecan-2	mAb (F-5), Ms IgG1κ<br>Santa Cruz Biotechnology, Cat#: sc-376160
	Syndecan-3	mAb (G-2), Ms IgMκ<br>Santa Cruz Biotechnology, Cat#: sc-398194
	Syndecan-4	pAb, Rb IgG<br>Abcam, Cat#: ab24511
	Glypican-1	mAb (A-10), Ms IgG1k<br>Santa Cruz Biotechnology, Cat#: sc-365000
	Glypican-2	mAb (F-5), Ms IgG1λ<br>Santa Cruz Biotechnology, Cat#: sc-393824
	Glypican-3	mAb (F-3), Ms IgM<br>Santa Cruz Biotechnology, Cat#: sc-390587
	Glypican-4	pAb, Rb IgG<br>Invitrogen, Cat#: PA5-88766
	Glypican-5	mAb (F-3), Ms IgG1κ<br>Santa Cruz Biotechnology, Cat#: sc-390838
	Glypican-6	pAb, Rb IgG<br>Biorbyt, Cat#: orb539585
	Perlecan	pAb, Rb IgG<br>Gift from Prof. Dziadek, University of NSW, Australia.
	Collagen XVIII / endostatin	Ms IgG2b (1837-46)<br>Santa Cruz Biotechnology, Cat#: sc-32720
	Agrin	Ms IgMκ (D2)<br>Santa Cruz Biotechnology, Cat#: sc-374117

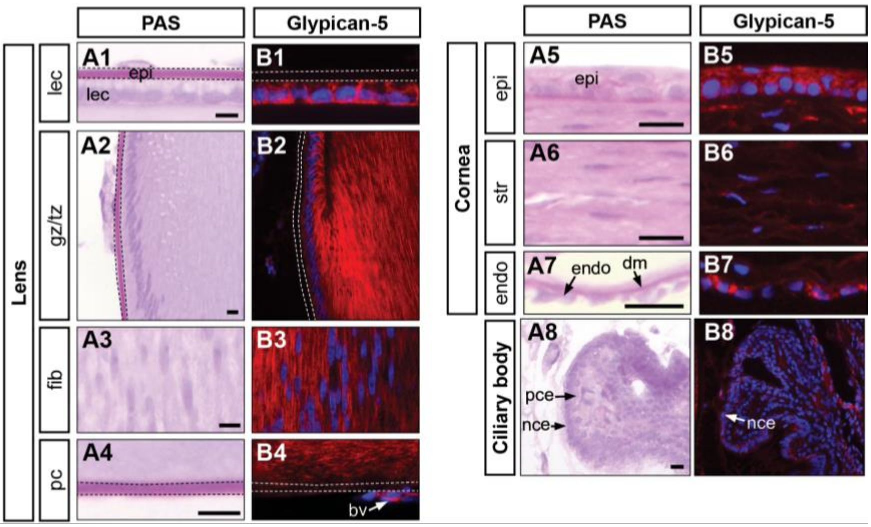

Supplementary Figure S1. Distribution of glypican-5 core protein in the 10-day-old rat eye. Mid-sagittal 10-day-old rat eye sections stained with Periodic acid-Schiffs (PAS, A1-8) or immunolabelled for Glypican-5 core protein (B1-8). Dotted white lines demarcate outline of the lens capsule. For immunofluorescence, nuclei were counterstained with Hoechst dye (blue). Abbreviations: Lens; central anterior epithelium (epi) and capsule (ac), germinative zone (gz), transitional zone (tz), lens fibre cells (fib), posterior lens capsule (pc), blood vessel (bv). Cornea; epithelium (epi), Bowman's capsule (bc), stroma (str), endothelium (endo), Descemet's membrane (dm). Ciliary body; non-pigmented ciliary epithelium (nce), pigmented ciliary epithelium (pce). Scale bars = 25 μm.
